# Spontaneous regression of Merkel cell carcinoma with positive detection of Merkel cell polyomavirus by PCR and immunohistochemistry^☆^

**DOI:** 10.1016/j.abd.2021.04.018

**Published:** 2022-12-09

**Authors:** Thiago Rubim Bellott, Flávio Barbosa Luz, Rafael Brandão Varella, Mayra Carrijo Rochael

**Affiliations:** a Department of Pathology, Universidade Federal Fluminense, Niterói, RJ, Brazil; b Department of Dermatology, Universidade Federal Fluminense, Niterói, RJ, Brazil; c Department of Microbiology and Parasitology, Universidade Federal Fluminense, Niterói, RJ, Brazil

Dear Editor,

Merkel cell carcinoma (MCC) is a rare cutaneous neoplasm, characterized by the proliferation of anaplastic cells, with an aggressive clinical course. It is more frequently diagnosed in caucasian males after the seventh decade of life and in immunosuppressed individuals.^1^

In 2008, Feng et al. observed the DNA of a new polyomavirus in 8 of 10 MCCs, named Merkel cell polyomavirus (MCPyV). The viral DNA was integrated into the DNA of the tumor cells in a clonal pattern, suggesting that the viral infection preceded the clonal expansion of these cells.^2^

A 76-year-old patient reported fast-growing nodules on the leg, with eight weeks of evolution. The physical examination showed a firm, erythematous, semispherical nodule measuring 4 cm on the left leg, surrounded by similar satellite lesions (Fig. 1A). These findings regressed considerably three weeks after a shave biopsy of the main lesion was performed (Fig. 1B).

**Figure 1:**
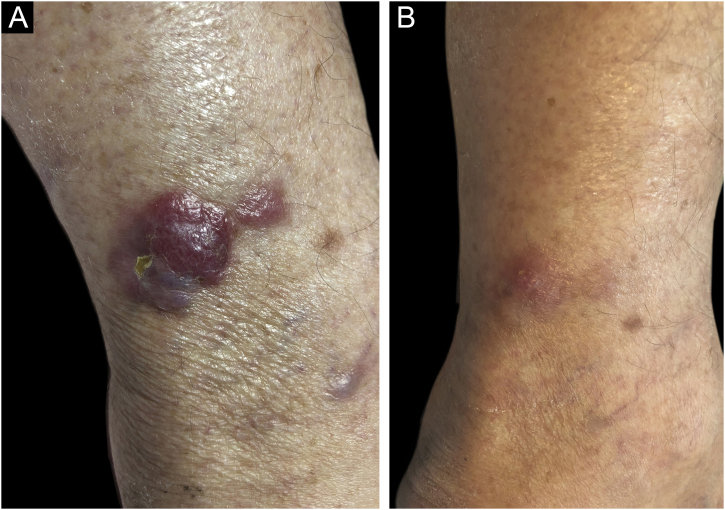
Tumor lesion on the left leg, before and after the shave biopsy. (A) A red, firm, 4-cm nodule on the left leg, with adjacent similar papules, before the shave biopsy. (B) Clinical aspect of tumor regression three weeks after the shave biopsy.

Histopathological analysis showed a dermal tumor with extensive proliferation of small basophilic cells, with large, ovoid, hyperchromatic nucleoli and finely dispersed chromatin (Fig. 2A). Immunohistochemistry was positive for CK20, with a perinuclear, dot-like pattern, and chromogranin A (Fig. 2B,C), and negative for TTF-1 and CK7, confirming the diagnosis of MCC. MCPyV DNA was detected by PCR and the major viral T-antigen was detected by nuclear positivity in immunohistochemistry using CM2B monoclonal antibody (Fig. 2D). Despite the observed partial regression, surgical excision was performed with wide margins, and there was no recurrence of the condition after two years of follow-up. Histopathology of the surgical specimen revealed residual neoplasia circumscribed by connective tissue strands and dermal fibrosis.

**Figure 2:**
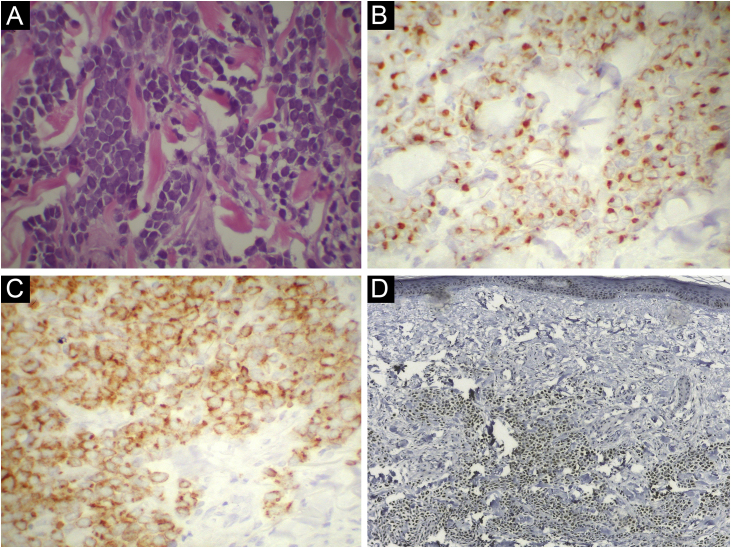
Histopathological and immunohistochemical staining. (A) Proliferation consisting of small basophilic cells, with large, ovoid, hyperchromatic nucleoli, and finely dispersed chromatin (Hematoxylin & eosin, ×400). (B) Immunohistochemical reactivity for CK20 in a perinuclear dot-like pattern, ×400. (C) Immunohistochemical reactivity for chromogranin A, ×400. (D) Immunohistochemical nuclear reactivity for MCPyV (CM2B monoclonal antibody), ×40.

Although there is no standard protocol, the treatment is based on the excision with wide margins for localized or locoregional disease, with adjuvant radiotherapy for large tumors. When there are distant metastases, radiotherapy and adjuvant chemotherapy are combined.^1^

The pathogenesis of MCC is considered multifactorial. Studies have reported a P53 mutation and high levels of bcl2 proto-oncogene expression in tumor cells, supporting rapid tumor expansion and growth.^1^, ^3^

Spontaneous regression of MCC is rare and was described in 1986, with fewer than 40 similar cases being reported since then. Regressions after biopsy or incomplete excision have also been described and may be due to the activation of the T-cell-mediated immune response after surgical trauma, although the exact mechanisms remain unknown.^4^

Unlike melanoma cases, the reported cases of MCC with spontaneous regression usually had a better prognosis and progressed to cure.^5^

The presence of MCPyV in MCC is thought to stimulate the triggering of an immune response against viral antigens and tumor cells.^5^ Considering the presence of MCPyV in the present report, it is postulated that viral antigen exposure after the biopsy may have triggered host immune activation and tumor regression.

In conclusion, the present report aims to draw attention to the rare possibility of spontaneous regression of MCC and its association with MCPyV.

## Financial support

FUNADERM (*Fundo de Apoio à Dermatologia*) in 2019.

## Authors' contributions

Thiago Rubim Batista Bellott Nascimento: Design and planning of the study; drafting and editing of the manuscript; collection, analysis, and interpretation of data; intellectual participation in the propaedeutic and/or therapeutic conduct of the studied cases; critical review of the literature; critical review of the manuscript.

Flávio Barbosa Luz: Approval of the final version of the manuscript; effective participation in research orientation; critical review of the manuscript.

Rafael Brandão Varella: Approval of the final version of the manuscript; collection, analysis, and interpretation of data; critical review of the manuscript.

Mayra Carrijo Rochael: Approval of the final version of the manuscript; effective participation in research orientation; critical review of the manuscript.

## Conflicts of interest

None declared.
